# Acacetin as a natural cardiovascular therapeutic: mechanisms and preclinical evidence

**DOI:** 10.3389/fphar.2025.1493981

**Published:** 2025-04-04

**Authors:** Zihe Zhou, Tao Li, Helin Qin, Xinyu Wang, Shanshan He, Zhongcai Fan, Qiang Ye, Yanfei Du

**Affiliations:** ^1^ Department of Cardiology, The Affiliated Hospital, Southwest Medical University, Luzhou, Sichuan, China; ^2^ Department of Clinical Medicine, School of Clinical Medicine, Southwest Medical University, Luzhou, Sichuan, China; ^3^ Key Laboratory of Medical Electrophysiology, Ministry of Education and Medical Electrophysiological key Laboratory of Sichuan Province, Institute of Cardiovascular Medicine, Southwest Medical University, Luzhou, Sichuan, China; ^4^ Department of Basic Medicine, School of Basic Medical Science, Southwest Medical University, Luzhou, Sichuan, China

**Keywords:** acacetin, flavonoids, cardiovascular disease, cardiovascular protective effects, potential mechanisms

## Abstract

Globally, cardiovascular disease (CVD) has emerged as a leading cause of mortality and morbidity. As the world’s population ages, CVD incidence is on the rise, and extensive attention has been drawn to optimizing the therapeutic regimens. Acacetin, a natural flavonoid derived from various plants, has been demonstrated to have a wide spectrum of pharmacological properties, such as antioxidant, anti-inflammatory, anti-bacterial, and anti-tumor activities, as well as protective effects on diverse tissues and organs. Recently, increasing numbers of studies (mostly preclinical) have indicated that acacetin has potential cardiovascular protective effects and might become a novel therapeutic strategy for CVDs. The importance of acacetin in CVD treatment necessitates a systematic and comprehensive review of its protective effects on the cardiovascular system and the underlying mechanisms involved. Here, we first provide an overview of some basic properties of acacetin. Subsequently, the protective effects of acacetin on multiple CVDs, like arrhythmias, cardiac ischemia/reperfusion injury, atherosclerosis, myocardial hypertrophy and fibrosis, drug-induced cardiotoxicity, diabetic cardiomyopathy, hypertension, and cardiac senescence, are discussed in detail. The underlying mechanisms by which acacetin exhibits cardiovascular protection appear to involve suppressing oxidative stress, reducing inflammation, preventing cardiomyocyte apoptosis and endothelial cell injury, as well as regulating mitochondrial autophagy and lipid metabolism. Meanwhile, several critical signaling pathways have also been found to mediate the protection of acacetin against CVDs, including phosphoinositide 3-kinase/protein kinase B/mechanistic target of rapamycin (PI3K/Akt/mTOR), sirtuin 1/AMP-activated protein kinase/peroxisome proliferator-activated receptor-γ coactivator-1α (Sirt1/AMPK/PGC-1α), transforming growth factor-β1/small mothers against decapentaplegic 3 (TGF-β1/Smad3), protein kinase B/endothelial nitric oxide synthase (Akt/eNOS), and others. Finally, we highlight the existing problems associated with acacetin that need to be addressed, such as the requirement for clinical evidence and enhanced bioavailability, as well as its potential as a promising cardiovascular drug candidate.

## 1 Introduction

Cardiovascular disease (CVD), consisting of heart and blood vessel conditions such as arrhythmia, cardiomyopathy, atherosclerosis, heart failure, and hypertension, remains the leading cause of death and long-term disability worldwide, and it also places a heavy burden on global health and the economy (Timmis et al., 2022; Benjamin et al., 2018). It is reported that about a third of deaths worldwide are closely associated with CVD. Despite significant progress in preventing and treating CVD, CVD is steadily increasing in incidence and mortality rates because of population growth and aging (Roth et al., 2015). Currently, the clinical management of CVD consists mainly of surgery and medication, but surgery is both risky and expensive. The existing drugs, such as β-blockers, statins, aspirin, and ACE inhibitors, are effective in treating CVD (Gao and Hou, 2023), but most of them may cause significant adverse effects with long-term use. Consequently, it is imperative to identify safe and effective preventive and therapeutic strategies to block or slow the progression of CVD.

**FIGURE 1:**
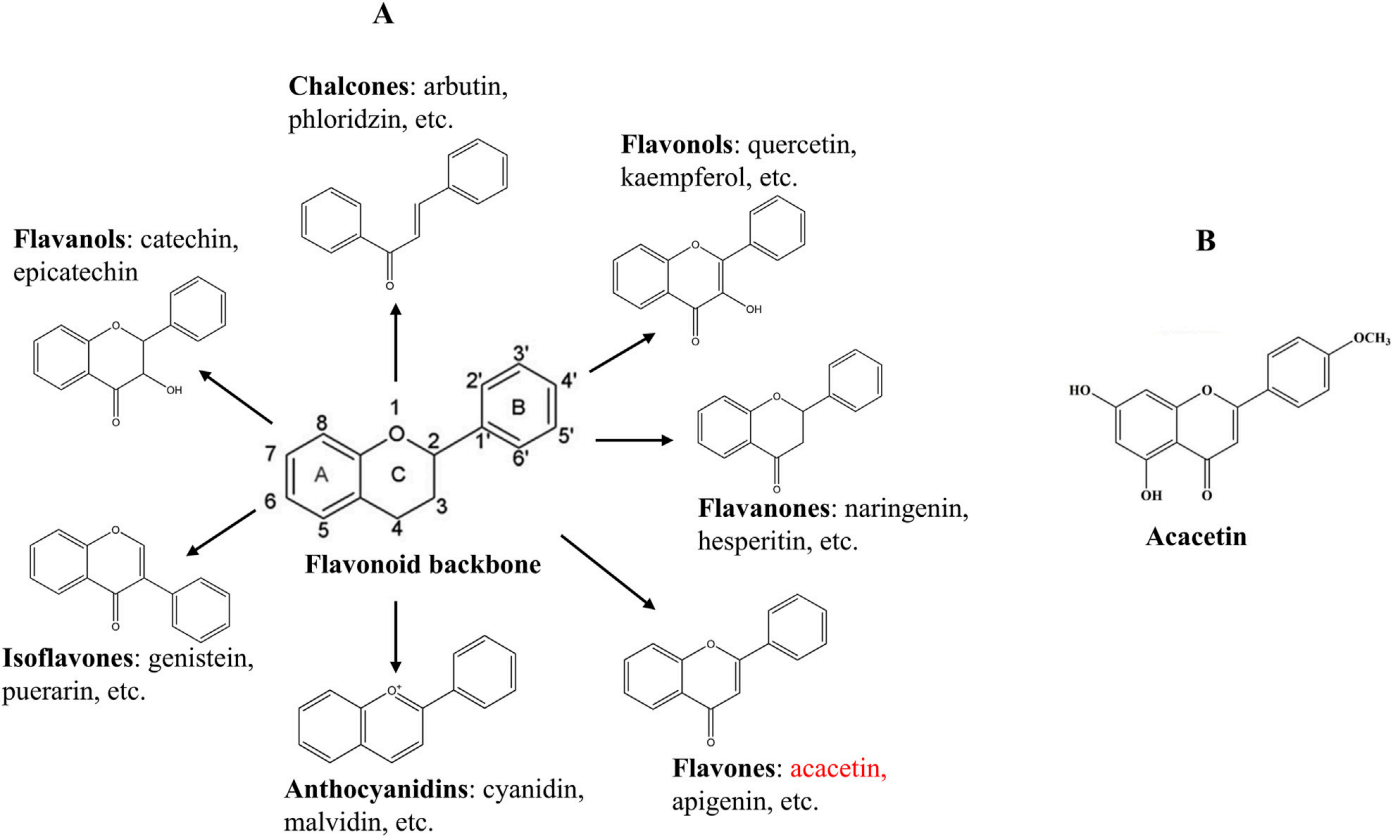
Chemical structures of the flavonoid backbone and its main subgroups **(A)**, and acacetin **(B)**.

For centuries, numerous traditional Chinese medicinal herbs have been broadly used to combat CVD in clinical settings, such as the representative radix astragalus used for heart failure (Yang et al., 2012), salvia miltiorrhiza applied in myocardial infarction (MI), angina pectoris, and stroke (Ji et al., 2000), and dragon’s blood used for acute MI (Li et al., 2018). Compared to conventional therapies that are expensive and frequently cause adverse reactions in the human body (Wang J. et al., 2024), traditional Chinese medicine remedies are becoming increasingly popular and receiving widespread attention due to their fewer side effects, lower cost and toxicity, and unique clinical efficacy in the treatment of CVD (Dai et al., 2024). Recently, more and more natural bioactive compounds extracted from traditional Chinese medicine have been found to exert potential therapeutic effects against CVD, like the well-known flavonoids (Leuci et al., 2020; Netala et al., 2024). Flavonoids are a large class of phenolic compounds widely present in plants and their basic skeleton (C6-C3-C6) is composed of a benzene ring A attached to a pyrone ring C and a phenyl ring B at position 2 or 3 (Figure 1A). They can be generally classified into seven distinct subtypes according to the structural differences, containing chalcones, isoflavones, flavones, flavanols, anthocyanidins, flavanones, and flavonols (Figure 1A). Studies have suggested that flavonoids possess various biological activities such as anticancer, antioxidant, and anti-inflammatory abilities and have the potential to treat diabetes, cancer, and CVDs (Panche et al., 2016; Zhao et al., 2019). Therefore, flavonoids are promising as novel candidate drugs due to their beneficial roles in disease prevention and treatment.

Acacetin (5,7-dihydroxy-4′-methoxyflavone, Figure 1B), as a flavone, exists naturally in various plants in the form of an aglycone or a glycoside, including Chrysanthemum, Robinia pseudoacacia, and Safflower (Singh et al., 2020). So far, acacetin has been demonstrated to possess diverse pharmacological effects, comprising antimicrobial, anti-inflammatory, anticancer, antiviral, antioxidant, and anti-infective functions, as well as protective effects against the nerves, heart, liver, and lungs (Singh et al., 2020; Wu et al., 2022b). Recently, increasing evidence has reported that acacetin exhibits potential curative effects on multiple CVDs with nearly no side effects, such as arrhythmia, myocardial hypertrophy, myocardial fibrosis (MF), drug-induced cardiotoxicity, atherosclerosis, cardiac aging, ischemia-reperfusion injury, and diabetic cardiomyopathy (DCM) (Chang et al., 2017; Wu et al., 2022b). Mechanistically, the cardiovascular protective functions of acacetin might be in association with modulating oxidative stress, inflammation, autophagy, cardiomyocyte apoptosis, cardiac fibroblast proliferation, lipid metabolism, and endothelial cell injury, involving many signaling cascades like phosphoinositide 3-kinase (PI3K)/protein kinase B (Akt)/mechanistic target of rapamycin (mTOR), sirtuin 1 (Sirt1)/AMP-activated protein kinase (AMPK)/peroxisome proliferator-activated receptor (PPAR)-γ coactivator-1α (PGC-1α), transforming growth factor-β1 (TGF-β1)/small mothers against decapentaplegic 3 (Smad3), Akt/endothelial nitric oxide synthase (eNOS), and others. This article presents an overall review of the protective effects of acacetin on different types of CVDs, highlights the underlying molecular mechanisms, and discusses the current problems and challenges before it becomes a clinical cardiovascular drug.

## 2 Properties of acacetin

### 2.1 Physicochemical properties of acacetin

As a flavone, acacetin is found naturally in up to 92 plants, especially abundant in chrysanthemum, turnera diffusa, safflower, propolis, linaria, betula pendula, and calamintha (Singh et al., 2020). Acacetin has a chemical formula of C_16_H_12_O_5_, and its relative molecular weight is 284.26 (Kim et al., 2014). It naturally exists in free or complex form (glycosylated compounds) in nature (Yang et al., 2014). The log P and log D_7.4_ values of acacetin exceed 3, suggesting that it is a highly lipophilic compound (Kiani and Jabeen, 2020; Hughes et al., 2008). Flavonoids generally exhibit poor water solubility, which may be a primary factor contributing to their limited oral bioavailability. For example, the double bonds between the C2 and C3 positions of flavones and flavonols make the molecular arrangement so compact that it is difficult for solvents to penetrate, thus leading to their poor solubility (Zhao et al., 2019). Previous studies showed that the oral bioavailability of the flavonol myricetin in rats was only 9.62%, which may be due to its limited water solubility of 16.60 μg/mL (Dang et al., 2014; Yao et al., 2014). Like other flavonoids, acacetin has a very low water solubility of 64.4 ± 10.9 ng/mL, and its solubility in ethanol is also limited to 0.712 ± 0.002 mg/mL (Wang et al., 2023a). Furthermore, acacetin was found to be stable under basic conditions but unstable under acidic and neutral conditions (Han et al., 2021).

### 2.2 Biosynthesis of acacetin

Because acacetin exists in many plants, traditional plant isolation was the main method of obtaining it in the past. The conventional method for the synthesis of acacetin was mainly based on the Baker-Venkataraman rearrangement (Pandurangan, 2014). In another method, Zhao et al. synthesized acacetin using 4-dimethylaminopyridine as a catalyst (Zhao et al., 2016). In addition, acacetin is also synthesized from naringenin chalcone, which requires chalcone isomerase, flavone synthase, and apigenin 4′-O-methyltransferase as catalysts (Liu, 2023). However, due to the low growth rates of plants and the time-consuming and complex process of plant extraction, researchers wanted to perform the heterologous biosynthesis of acacetin. Microbial co-culture technology made the idea a reality. In comparison to traditional plant extraction, microbial production has the advantages of low cost, high yield, sustainability, and environmental friendliness (Dudnik et al., 2018). Recently, Wang et al. achieved the first *de novo* biosynthesis of acacetin in a heterologous microbial host by designing and using a two- or three-strain *E. coli* co-culture system, which could convert simple carbon substrate glucose to the final product acacetin (Wang et al., 2020). Importantly, they discovered that cultivating the three-strain co-culture in shake flasks resulted in the maximum production of acacetin at 20.3 mg/L after 48 h (Wang et al., 2020).

### 2.3 Pharmacokinetics and delivery strategy of acacetin

In previous studies, data showed that the bioavailability of acacetin in FVB mice was only 1.3% (Jiang et al., 2017), and the maximum plasma concentration (*C*_max_) of acacetin in rats was 19.02 ± 1.29 ng/mL following oral administration of 6 mL/kg acacetin crude extract (Zhang et al., 2014). Meanwhile, the concentration of acacetin in rat plasma reached its peak 5 min after oral administration (Zhang et al., 2014). Another study suggested that after oral delivery of 100 mg/kg acacetin in rats, the unabsorbed dose of acacetin in the jejunal segments amounted to 97.1%, whereas the absorbed dose was only 2.34% (Han et al., 2021). In addition, Fan et al. conducted a pharmacokinetic study of acacetin in rats using a sensitive and rapid ultra-performance liquid chromatography-tandem mass spectrometry (UPLC-MS/MS) method, and the results indicated that after a single intravenous administration of acacetin at the dose of 5.0 mg/kg, the mean *C*_max_ of acacetin reached 1334.9 ± 211.6 ng/mL, succeeded by a rapid decrease in the blood concentration with a terminal half-life of 1.48 ± 0.53 h (Fan et al., 2015). Similarly, Kim and colleagues developed a highly selective LC-MS/MS method to perform the quantitative bio-analysis of acacetin in human plasma, and the results showed that the average recovery of acacetin in human plasma ranged from 91.5% to 95.6%, and the fraction of unbound acacetin in plasma was less than 1% (Kim et al., 2016). Acacetin is extensively metabolized by many tissues in the body, especially the liver. A systematic study on acacetin metabolism in rats revealed that the major phase I metabolic reaction of acacetin is an oxidation reaction principally catalyzed by cytochrome P450 enzymes, and about 10 phase I metabolites were identified, including luteolin, naringenin, and apigenin (Yin et al., 2019; Hodek et al., 2002). Furthermore, 21 metabolites of the phase II response of acacetin were recognized in rats, primarily comprising monosulfate and monoglucuronide produced mostly by UDP-glucuronosyltransferase 1A8 and sulfotransferase 1A1, respectively (Yin et al., 2019; Zhang et al., 2017; Dai et al., 2015).

As mentioned above, like many natural flavonoids, acacetin has several disadvantages, such as poor water solubility, rapid metabolism, and low bioavailability, which greatly limit its therapeutic potential. To address these issues, many promising strategies, like nanoparticle formulations, chemical modifications, microemulsions, and absorption enhancers, have been employed to increase the water solubility and gastrointestinal tract absorption of acacetin. For example, Wang et al. developed a novel acacetin-loaded microemulsion formulation that could significantly improve the solubility and percutaneous absorption efficiency of acacetin when combined with appropriate penetration enhancers (Wang et al., 2023a). Liu et al. successfully synthesized an acacetin prodrug through the structural modification of acacetin, namely, acacetin phosphate sodium, which could increase the water solubility of acacetin by over 1.9 million times and is suitable for intravenous administration (Liu et al., 2016a).

### 2.4 Toxicity of acacetin

Although studies on the toxicity of acacetin are limited, current research indicates that its consumption does not result in significant adverse effects and may be considered safe for medical use. The results of toxicity studies on acacetin are summarized in Table 2. The acute toxicity of acacetin was not observed in mice orally administered a maximum dose of acacetin (900 mg/kg, administered in three doses of 300 mg/kg each within 3 h), and no mortality or abnormal activity was observed in any animal during 2 weeks of observation (Li et al., 2008; Wang S. Y. et al., 2024). To evaluate acacetin prodrug’s acute toxicity, researchers administered a single dose of acacetin prodrug to mice via tail vein injection and found that the median lethal dose (LD_50_) was 721.7 mg/kg (Liu et al., 2016a). After converting the drug dosage based on body surface area (Blanchard and Smoliga, 2015), the LD_50_ of the acacetin prodrug in dogs was 108.3 mg/kg, which was far more than the median effective dose in dogs with AF (Liu et al., 2016a). In addition, no subacute toxicity was observed in mice intraperitoneally administered 400 mg/kg of the acacetin prodrug daily for a total of 14 days (Wang S. Y. et al., 2024). Previous studies showed that acacetin did not exhibit significant toxicity in normal lung fibroblasts (WI-38 cells) treated with a high concentration of 50 μM (Chien et al., 2011) or in bone marrow-derived macrophages treated with the concentration range of 0–20 μM (Lin et al., 2022). Also, acacetin was found to have no adverse effects on seminiferous tubules of male Balb/c mice in any dose tested (Ghanbari et al., 2022). Additionally, it showed no insecticidal activity against *Aedes atropalpus* mosquito larvae (Pereda-Miranda et al., 1997). Acacetin (<50 mg/kg) was demonstrated to have no obvious toxic side effects on the liver and kidney in prostate tumor-bearing nude mice at the doses tested (Kim et al., 2014). Even with prolonged use or at a high dose (50 mg/kg), acacetin exerted no detrimental impact on the urinary as well as reproductive systems in mice (Shokri et al., 2020).

**TABLE 2:** The summary of toxicity studies on acacetin.

Type and dose of acacetin	Test model/object	Administration route and/or duration of action	Result	Refs
Acacetin, 25, 50 mg/kg	The liver and kidney of nude mice bearing DU145 tumor xenografts	Oral administration, 3 times a week for total 7 weeks	No significant toxicity	Kim et al. (2014)
Acacetin, 10, 25, 50 mg/kg/day	The reproductive and urogenital systems of male Balb/c mice	Intraperitoneal injection, 3 days and 10 days	No significant toxicity	Shokri et al. (2020)
Acacetin, 10, 25, 50 mg/kg/day	The seminiferous tubules of male Balb/c mice	Intraperitoneal injection, 3 days and 10 days	No significant toxicity	Ghanbari et al. (2022)
Acacetin prodrug, 540–900 mg/kg	ICR mice	Single intravenous injection, 24 h	Obtaining the LD_50_ value of 721.7 mg/kg	Liu et al. (2016a)
Acacetin prodrug, dose conversation	Dogs	Intravenous injection	Predicting the LD_50_ value of 108.3 mg/kg	Liu et al. (2016a)
Acacetin prodrug, 400 mg/kg/day	Mice	Intraperitoneal injection, 14 days	No subacute toxicity	Wang et al. (2024b)
Acacetin, 900 mg/kg	Mice	Oral administration, 300 mg/kg each time and 3 times within 3 h, 14 days	No acute toxicity and no animal deaths	Li et al. (2008)
The CHCl_3_-soluble extract of Piperaceae	*Aedes atropalpus* mosquito larvae	-	No insecticidal activity	Pereda-Miranda et al. (1997)
Acacetin, 0–50 μM	WI-38 cells	24 h and 48 h	No significant cytotoxicity	Chien et al. (2011)
Acacetin, 0–20 μM	Bone marrow-derived macrophages	24 h and 48 h	No significant cytotoxicity	Lin et al. (2022)

### 2.5 Bioactivities of acacetin

In previous reports, acacetin is known to be a potent molecule possessing extensive pharmacological potential, such as the most common anti-apoptotic, anti-inflammatory, antioxidant, and anti-tumor actions. Due to these pharmacological properties, acacetin shows protective and therapeutic effects on various diseases, including cancer, liver diseases, neurological disorders, and CVDs. The underlying mechanisms of acacetin protecting against these diseases may involve the regulation of diverse signaling pathways, including mitogen-activated protein kinases (MAPK), c-Jun N-terminal kinase (JNK), extracellular signal-regulated kinase (ERK), nuclear factor-kappa B (NF-κB), nuclear factor erythroid 2-related factor 2 (Nrf2), phosphoinositide 3-kinase (PI3K)/protein kinase B (Akt)/mechanistic target of rapamycin (mTOR), and cyclooxygenase-2 (COX-2) signaling (Singh et al., 2020).

#### 2.5.1 Anti-oxidant activity

Based on the tricyclic structure of flavonoids, the phenolic hydroxyl group in acacetin can react with oxygen radicals to form stable semiquinone radicals, thus terminating free radical chain reactions and exerting antioxidant activity (Sun et al., 2012). Studies have demonstrated that acacetin exerts antioxidant activity via removing free radicals, increasing antioxidant protein expression, and suppressing the activity of free radical-generating enzymes. In mouse liver homogenate, acacetin was identified to prevent lipid peroxidation and scavenge superoxide anions (Chen et al., 1990). In a rat model of myocardial ischemia/reperfusion (I/R) injury, the cardioprotective effect of the acacetin prodrug was partially attributed to its ability to prevent the reduction of antioxidant proteins superoxide dismutase 2 (SOD2) and thioredoxin (Liu et al., 2016b). *In vitro*, acacetin could suppress the production of 1,1-diphenyl-2-picrylhydrazyl (DPPH) free radicals and obviously mitigate the lipid peroxidation (Sun et al., 2012). In addition, another study reported that in hypoxia/reoxygenation (H/R)-treated neonatal rat cardiomyocytes, acacetin attenuated cell injury through reduction of lipid peroxidation and upregulation of antioxidant protein expression (Yang et al., 2014). Recently, acacetin was shown to prevent renal I/R damage in mice by markedly reducing the level of malondialdehyde (MDA) by approximately 70% and enhancing the total antioxidant capacity in kidney tissue (Shiravi et al., 2020).

#### 2.5.2 Anti-inflammation

Acacetin has been shown to prevent inflammation in several studies, thus delaying or improving many diseases, such as sepsis-induced acute lung injury (Chang et al., 2024), ulcerative colitis (Ren et al., 2020), pancreatic and hepatorenal dysfunction associated with type 2 diabetes (Wang et al., 2023b), and Parkinson’s disease (Kim et al., 2012). In an animal model of cerebral I/R injury, acacetin could decrease the release of pro-inflammatory cytokines, like tumor necrosis factor-α (TNF-α), interleukin (IL)-6, and IL-1β, through regulating the toll-like receptor 4 (TLR-4)/NF-κB/nucleotide-binding oligomerization domain (NOD)-, leucine-rich repeat (LRR)-, and pyrin domain (PYD)-containing protein 3 (NLRP3) signaling axis, demonstrating a neuroprotective role (Bu et al., 2019). Besides, several studies reported that the production and activity of other pro-inflammatory mediators including COX-2, 5-lipoxygenase, NO, prostaglandin E2, leukotriene B4, and 5-hydroxyeicosatetraenoic acid could also be suppressed by acacetin in numerous types of cells (Fan et al., 2012; Kim et al., 2012; Srisook et al., 2015; Liao et al., 1999). Acacetin has also been shown to alleviate inflammation by preventing NLRP3 inflammasome activation through the modulation of signal molecules such as NF-κB p65, p38 MAPK, ERK, and JNK in mouse bone marrow-derived macrophages (Bu et al., 2024). Moreover, acacetin was identified to reduce gastrointestinal inflammation, probably through regulating the PI3K/Akt signaling cascade (Guo et al., 2021).

#### 2.5.3 Anti-cancer

Acacetin has demonstrated anti-cancer potential in many cancer cell lines, which may be closely related to its antiproliferative and anti-invasive effects mediated by the activation of the NF-κB and MAPK pathways and the phosphorylation of Akt. In hepatocellular carcinoma HepG2 cells, T-cell leukemia Jurkat cells, and human non-small cell lung cancer A549 cells, Acacetin could significantly promote apoptosis of these cells, and the mechanism may be related to the Fas-mediated pathway (Watanabe et al., 2012; Hsu et al., 2004). In another report, acacetin blocked 12-O-tetradecanoylphorbol-13-acetate (TPA)-evoked migration and invasion of A549 cells by inhibiting JNK phosphorylation and decreasing NF-κB and activator protein-1 binding activity (Fong et al., 2010). Also, in DU145 cells, acacetin was a strong inhibitor of NF-κB signaling, blocking the Akt/NF-κB signaling activation and thus suppressing cell proliferation while inducing cell apoptosis (Kim et al., 2014). Besides, acacetin caused the decline in mitochondrial membrane potential, promoted the release of cytochrome c from mitochondria, and induced cell apoptosis in a variety of tumor cells, which was closely correlated with the MAPK-mediated signaling pathway (Shim et al., 2007; Prasad et al., 2020; Pan et al., 2005; Kim et al., 2015). Notably, it was found that the inactivation of PI3K/Akt/mTOR/p70S6K/ULK cascade was crucial in acacetin-induced autophagy, cell cycle arrest, and apoptosis in human breast cancer cells (Zhang et al., 2018).

#### 2.5.4 Anti-apoptotic activity

As mentioned above, accumulating studies have demonstrated that the pro-apoptotic activity of acacetin takes a significant part in preventing and delaying tumor development. In addition, acacetin also has an anti-apoptotic ability and can provide protective effects on the nerves, liver, and especially the heart. In 6-hydroxydopamine-induced neuronal cells, acacetin exerted neuroprotective effects by inhibiting neurotoxicity and neuronal cell death through the prevention of oxidative stress, apoptotic pathways, and PI3K/Akt, p38 MAPK, JNK, and glycogen synthase kinase-3β (GSK-3β) phosphorylation (Kim et al., 2017). It was shown that a water-soluble acacetin prodrug could prevent acetaminophen-induced hepatocyte apoptosis and acute liver injury both *in vitro and in vivo*, likely through the activation of PPAR-γ signaling and the suppression of endoplasmic reticulum stress (Miao et al., 2023). In mice with MI, acacetin significantly reduced the levels of phosphorylated p65 (p-p65), Bax, and cleaved caspase-3 through regulating NF-κB signaling, thereby leading to a decrease in cardiomyocyte apoptosis after MI (Chang et al., 2017). In high glucose-exposed cardiomyocytes, acacetin mitigated high glucose-stimulated cell injury and apoptosis, which may be associated with the PPAR-α/AMP-activated protein kinase (AMPK) signaling activation (Song et al., 2022). Likewise, in high glucose-treated human umbilical vein endothelial cells (HUVECs), acacetin was found to protect against endothelial cell injury by enhancing cell viability, inhibiting apoptosis, and reducing oxidative stress, which was mediated by the activation of Sirt1/Sirt3/AMPK pathway (Han et al., 2020). Additionally, in cultured primary cardiomyocytes and H9C2 cells, acacetin obviously reduced H/R-evoked cell injury by blocking oxidative stress, apoptosis, and inflammation via the upregulation of the AMPK/Nrf2 signaling (Wu et al., 2018).

## 3 Cardiovascular actions of acacetin: pharmacological effects and molecular mechanisms

### 3.1 Anti-arrhythmias

#### 3.1.1 Anti-atrial fibrillation

Atrial fibrillation (AF) is the most common arrhythmic disorder and has become an increasingly significant clinical problem with advancing age (Staerk et al., 2017). Up to 33.5 million people worldwide are estimated to have AF (Sagris et al., 2021), and AF patients face a higher risk of stroke, heart failure, dementia, and death (Kirchhof et al., 2016). Mechanistically, electrical and structural remodeling of the atria is generally believed to have a crucial part in AF formation. Despite clinically antiarrhythmic drugs still being used as the first-line treatment for AF rhythm control, some of their side effects, such as long QT syndrome and lethal torsade de pointe (TdP), are evident in long-term use (Fuster et al., 2006). The available data show that several atrial-specific ion channel currents, such as the ultrarapid delayed rectifier K^+^ current (*I*_Kur_, encoded by the K^+^ channel Kv1.5), the small-conductance Ca^2+^-activated K^+^ current (*I*_SKCa_, encoded by the SK channels), and the acetylcholine-activated K^+^ current (*I*_KACh_), may be novel targets to develop atrial-selective anti-AF drugs (Al-Khatib et al., 2014; Burashnikov and Antzelevitch, 2010; Diness et al., 2010).

Several studies have demonstrated that acacetin has potential anti-AF effects, but these results are based only on *in vitro* and animal models and require further clinical validation. In one study on AF, acacetin (2.5–10 μM) was suggested to prolong the atrial effective refractory period (ERP) and prevent experimental AF formation in anesthetized dogs without increasing the QTc interval (Li et al., 2008). Additionally, acacetin was found to preferentially inhibit the *I*_Kur_ and transient outward K^+^ current (*I*_to_), as well as prolong the action potential (AP) duration in human atrial myocytes (Li et al., 2008). In this study, the authors also found that acacetin had an inhibitory effect on the *I*_KACh_, but not on the inward rectifier K^+^ current, L-type Ca^2+^ current, or Na^+^ current (*I*_Na_). Moreover, studies found that acacetin (3–30 μM) could predominantly block SK_Ca_ channels expressed in HEK 293 cells, in addition to inhibiting Kv1.5 and Kv4.3 channels, which might also be closely associated with the anti-AF potential of acacetin (Chen et al., 2017; Wu et al., 2011; Wu et al., 2013). Further studies revealed that acacetin (3–10 μM) blocked Kv1.5 and Kv4.3 channels by binding to the S6 region of Kv1.5 and the T366 and T367 residues in the p-loop helix, as well as to the V392, I395, and V399 residues in the S6 transmembrane domain of Kv4.3, respectively (Wu et al., 2011; Wu et al., 2013). Due to the low water solubility of acacetin, which may affect its efficacy in treating AF when administered intravenously, Li’s team synthesized a well-soluble acacetin prodrug, acacetin phosphate sodium, by coupling a polar phosphate group to the 7-OH of acacetin and demonstrated its ability to terminate experimental AF caused by atrial rapid pacing with vagal nerve stimulation in beagle dogs (Liu et al., 2016a). Recently, one study reported that the combined use of acacetin (3.2 μM) and sodium channel blockers could produce synergistic antiarrhythmic effects in chronic AF-remodeled human atria by jointly blocking the K^+^- and Na^+^-currents, without remarkably changing QT intervals and ventricular repolarization (Ni et al., 2017). This strategy appears to be an effective option for treating AF. The underlying anti-AF effects and mechanisms of acacetin mentioned above are summarized in Table 1.

**TABLE 1:** Anti-arrhythmic effects of acacetin and its underlying mechanisms of action.

Types of arrhythmia	Test model	Result and mechanism	References
Atrial fibrillation	Human or guinea pig atrial myocytes, HEK293 cells stably expressing hERG or *I*_Ks_ Channels, and an experimental AF model in anesthetized dogs	Inhibit *I*_Kur_ and *I*_to_ currents, prolong AP duration, repress carbachol-evoked *I*_KACh_ current, block *I*_hERG_ and *I*_Ks_ currents, prolong atrial ERP without causing QTc prolongation and prevent AF induction	Li et al. (2008)
	HEK293 cells stably expressing related iron channels such as SK_Ca_, hKv1.5, or hKv4.3 channels	Suppress SK_Ca_ channels by interacting with the P-loop helix, inhibit hKv1.5 channels by binding to the S6 domain, block hKv4.3 channels by binding to their P-loop helix and S6 domain	Chen et al. (2017), Wu et al. (2011), Wu et al. (2013)
	An AF model in beagle dogs induced by vagal nerve stimulation and burst atrial pacing, HEK293 cells expressing hKv1.5 or hKv4.3 gene, and rat atrial myocytes	Inhibit hKv1.5, hKv4.3, and *I*_KACh_ currents, terminate the experimental AF effectively	Liu et al. (2016a)
Ventricular fibrillation	Contemporary mathematical models of human atrial and ventricular myocytes, multicellular two- or three-dimensional anatomical models of the human atria	Produce synergistic anti-AF effects by simultaneously blocking the Na^+^- and K^+^-currents with combined use of Na^+^-blockers and acacetin	Ni et al. (2017)
	Isolated canine ventricular epicardial myocytes, wedge and whole-heart models of JWS	Decrease *I*_to_ density, AP notch, and J wave area and suppress the electrocardiographic and arrhythmic manifestations of JWS	Di Diego et al. (2020)
	Patient-derived iPSC-CMs with KCND3 gain-of-function variant	Inhibit *I*_to_ currents and reduce the accentuated AP notch	Ye et al. (2022)

#### 3.1.2 Anti-ventricular fibrillation

J-wave syndrome (JWS), mainly containing early repolarization syndrome (ERS) and Brugada syndrome (BrS), manifests as J-wave protrusion and/or ST-segment elevation in specific electrocardiogram leads and is often related to fatal ventricular tachycardia and fibrillation (Antzelevitch and Yan, 2015). In clinical practice, implantable cardioverter defibrillators (ICDs) and quinidine are commonly used to treat high-risk patients suffering from JWS (Antzelevitch et al., 2016). Nevertheless, ICDs have a higher incidence of complications and are inapplicable to infants and young children. As an alternative to ICD therapy, quinidine can effectively prevent ventricular fibrillation in BrS patients but may prolong QT intervals and cause fatal TdP (Belhassen et al., 2004). Recently, a research team demonstrated that acacetin (5–10 μM) significantly inhibited electrocardiographic and arrhythmic manifestations of BrS and ERS by decreasing the AP notch, *I*_to_ density, and J-wave area in a variety of experimental models of JWS (Table 1) (Di Diego et al., 2020). Similarly, Ye et al. indicated that acacetin may become a promising treatment for JWS caused by KCND3 gain-of-function mutations through repressing *I*_to_ and the AP notch in cardiomyocytes differentiated from patient-specific induced pluripotent stem cells (iPSCs) (Table 1) (Ye et al., 2022).

### 3.2 Reducing myocardial I/R injury

Ischemic cardiomyopathy (ICM) such as acute MI is one of the leading causes of death and disability worldwide. After the onset of ischemia, cardiac reperfusion to restore coronary and myocardial blood supply is usually considered as the most critical measure to save ischemic myocardium (Lejay et al., 2016). Clinically, thrombolysis, percutaneous coronary intervention, and coronary artery bypass grafting are the most commonly used revascularization techniques for ICM. However, reperfusion can cause more severe cardiac injury than ischemia alone, called myocardial I/R injury. Studies have indicated that various mechanisms, including oxidative stress, mitochondrial dysfunction, inflammation, autophagy, and cell apoptosis, are involved in cardiac I/R injury (Xiao et al., 2022; Liu et al., 2023). I/R injury is able to cause infarct expansion, arrhythmias, cardiac dysfunction, and even death (Rassaf et al., 2014). Therefore, developing novel therapies to reduce I/R injury is of particular importance.

**FIGURE 2:**
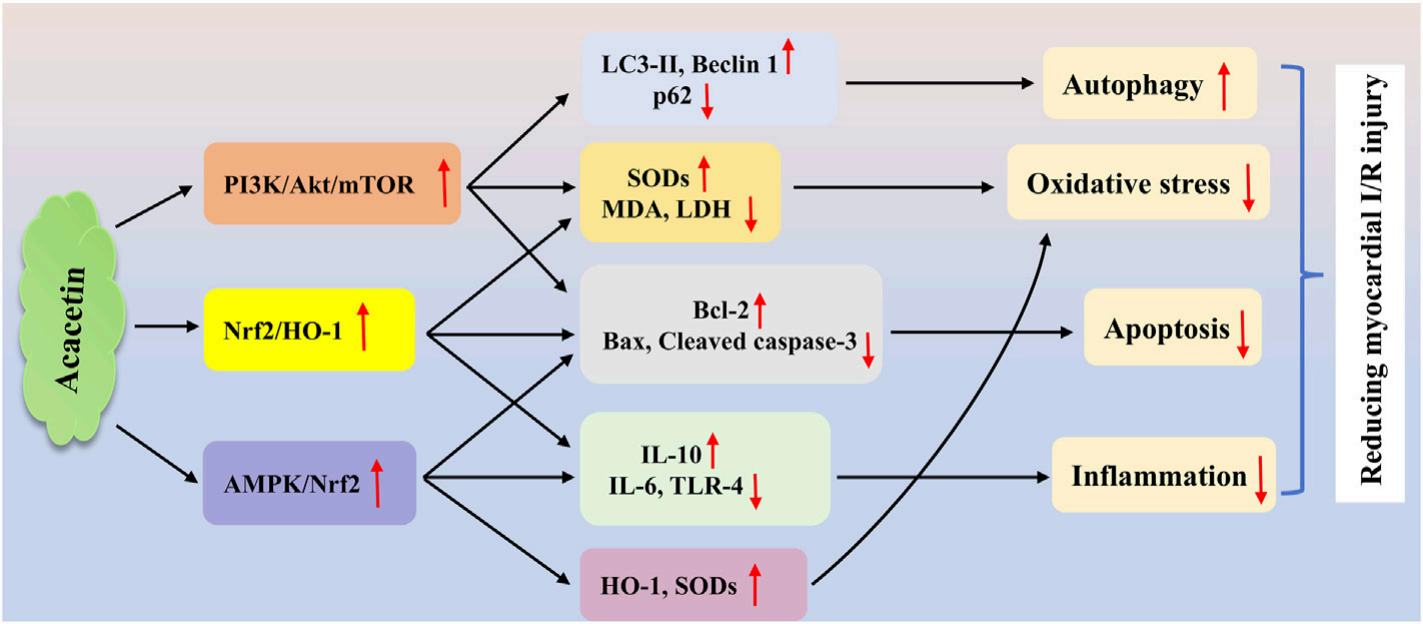
The potential mechanisms of acacetin in treating myocardial I/R injury. Acacetin protects against cardiac I/R injury mainly by inducing autophagy, reducing oxidative stress, and inhibiting inflammation and cell apoptosis via multiple signaling pathways, including the PI3K/Akt/mTOR (Liu C. et al., 2021), Nrf2/HO-1 (Wu et al., 2022c), and AMPK/Nrf2 (Wu et al., 2018) cascades. 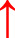: activation or upregulation; 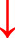: inhibition or downregulation.

In a H/R injury model of cardiomyocytes, which is often used to simulate cardiac I/R injury *in vitro*, acacetin significantly decreased lipid peroxidation and enhanced antioxidant activity in neonatal rat cardiomyocytes, thereby attenuating cellular H/R injury (Yang et al., 2014). Similarly, Wu et al. demonstrated that the protective effect of acacetin (0.3–3 μM) against H/R injury in cardiomyocytes was associated with the activation of AMPK/Nrf2 signaling, thereby reducing cell apoptosis and reactive oxygen species (ROS) production, restoring the levels of SOD1 and SOD2, preventing the secretion of TLR-4 and IL-6, and increasing the production of IL-10 (Wu et al., 2018). Furthermore, in H9C2 cardiomyocytes exposed to H/R insult, acacetin (12.5–50 μg/mL) protected against H/R-induced damage by enhancing autophagy through the activation of the PI3K/Akt/mTOR cascade (Liu C. et al., 2021). In an *in vivo* study, an acacetin prodrug (10 mg/kg) showed notable cardioprotective effects on I/R injury in rats (Liu et al., 2016b). Mechanistically, acacetin markedly inhibited the reduction of antioxidant levels and the expression of pro-inflammatory mediators, while preventing cardiomyocyte apoptosis, thereby exerting obvious cardioprotection against I/R injury. In another study on rat cardiac I/R injury, acacetin (10 mg/kg) attenuated cardiac I/R injury mainly by activating the Nrf2/heme oxygenase-1 (HO-1) signaling pathway to reduce inflammation, oxidative stress, and cardiomyocyte apoptosis (Wu et al., 2022c). The potential mechanisms of acacetin in reducing cardiac I/R injury are summarized in Figure 2.

### 3.3 Anti-atherosclerosis

Atherosclerosis emerges as a chronic progressive disease of the arteries, which is attributed to inflammatory reaction, oxidative stress, lipid dysregulation, and epigenetic disorders (Agrawal et al., 2020; Khosravi et al., 2019). It starts with endothelial injury, followed by oxidized low-density lipoprotein (ox-LDL) aggregation in the intima and vascular smooth muscle cell activation, ultimately leading to plaque instability and rupture, which can have fatal consequences (Wu et al., 2022c; Grootaert and Bennett, 2021; Koenig and Khuseyinova, 2007). Stroke, ICM, and peripheral arterial disease are the main clinical manifestations of atherosclerosis and collectively represent the leading cause of cardiovascular mortality (Libby and Theroux, 2005). Despite the good effects of current treatments, many patients still encounter serious coronary events (Libby, 2005). Therefore, the development of new therapeutic approaches for treating atherosclerosis is essential.

**FIGURE 3:**
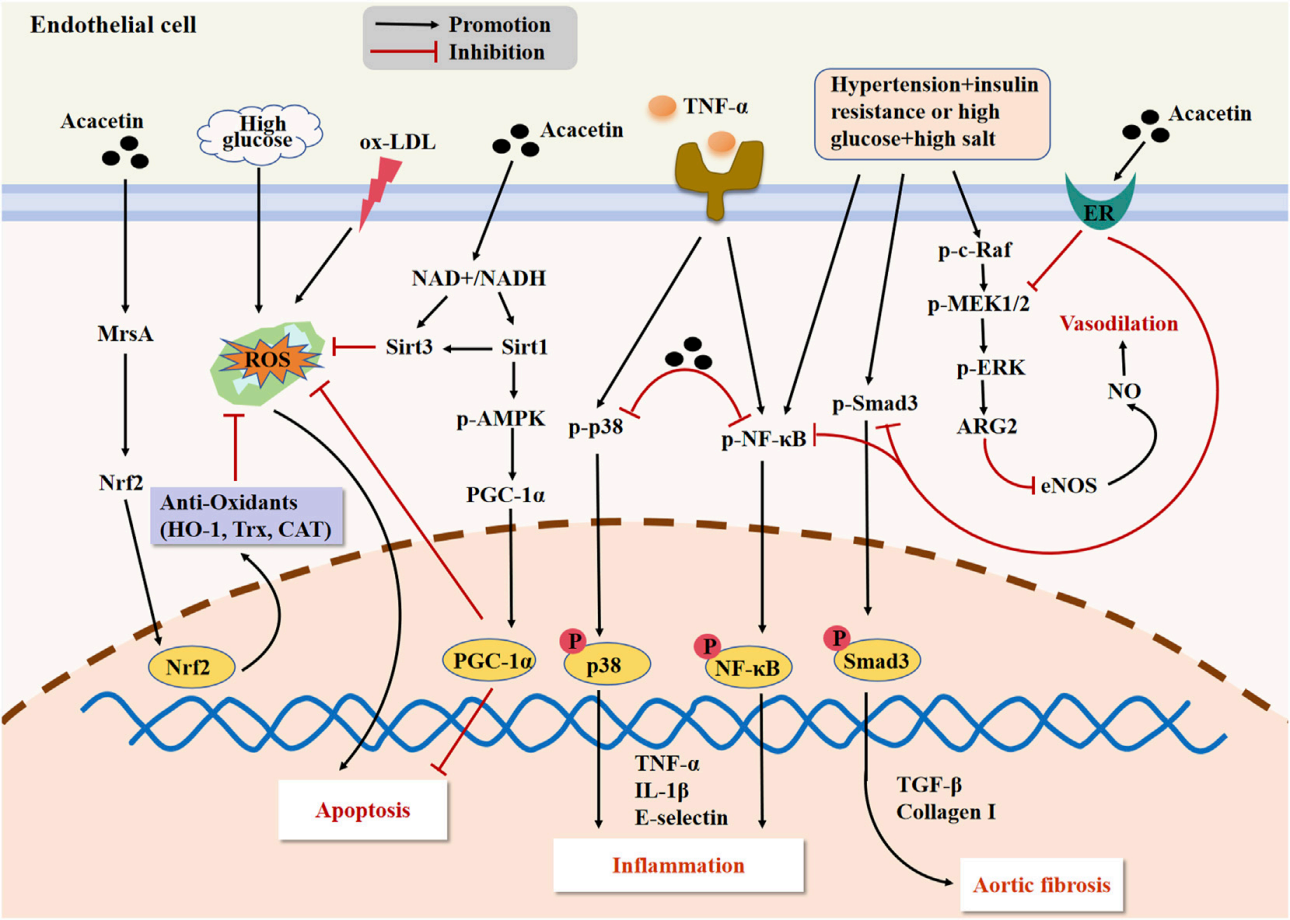
The major signaling pathways of acacetin in protecting against endothelial injury and improving atherosclerosis. Acacetin not only alleviates vascular inflammation by regulating the p38 MAPK and NF-κB pathways, but also restores mitochondrial function and reduces endothelial cell apoptosis through activating the Sirt1/AMPK/PGC-1α signaling. Acacetin can also activate the MsrA-Nrf2/Keap1 pathway to enhance cellular antioxidant capacity, ultimately reducing ox-LDL-induced endothelial cell apoptosis. Additionally, acacetin can activate the estrogen receptor (ER) pathway to suppress the expression of inflammatory factors and fibrosis-related genes while promoting the release of NO, thus improving endothelial dysfunction, vasodilation, and aortic fibrosis in SHR with insulin resistance.

Hyperlipidemia can promote atherosclerosis development through accelerating the formation and accumulation of ox-LDL in the subendothelial space. Obesity is an important cause leading to hyperlipidemia. A previous study demonstrated that the adipogenesis in adipocytes and lipid deposition in high-fat diet-evoked obese mice were notably reduced by acacetin, indicating acacetin’s potential anti-obesity effects (Liou et al., 2017). Endothelial dysfunction is recognized as a feature of many different human panvascular diseases, comprising diabetes, atherosclerosis, and hypertension (Xu et al., 2021). In this context, endothelial cells can serve as a potential interventional target for acacetin to improve endothelial function and prevent atherosclerosis. In an *in vitro* study, acacetin (30 μM) prevented TNF-α-induced E-selectin expression and monocyte-endothelial interaction in HUVECs partly through inhibiting the activation of p38 MAPK and NF-κB pathways, thus exerting its potential therapeutic value in vascular inflammation (Tanigawa et al., 2013). Wei et al. confirmed that acacetin (25–50 mg/kg) attenuated endothelial dysfunction and aortic fibrosis in spontaneous hypertension rats (SHR) with insulin resistance by suppressing inflammatory responses and improving vasodilatory function through the activation of estrogen receptors (Wei et al., 2020). Besides, Han et al. showed that acacetin (0.3–3 μM in vitro; 20 mg/kg in vivo) attenuated hyperglycemia-induced endothelial injury by restoring mitochondrial function through regulation of the Sirt1/AMPK/PGC-1α pathway, thus ameliorating diabetes-stimulated atherosclerosis in ApoE^−/−^ mice (Han et al., 2020). This study revealed that acacetin was expected to become a novel therapy for improving atherosclerosis in patients. Recently, another report revealed that acacetin (0.3–3 μM *in vitro*; 15 mg/kg *in vivo*) not only reduced ox-LDL-caused endothelial cell apoptosis by enhancing cellular antioxidant capacity through the methionine sulfoxide reductase A (MsrA)-Nrf2/Kelch-like ECH-associated protein 1 (Keap1) signaling axis *in vitro* but also markedly alleviated atherosclerosis by inhibiting oxidative stress and inflammation while promoting lipid metabolism in Western diet-fed ApoE^−/−^ mice, which also suggested a potential therapeutic effect of acacetin on atherosclerosis-related CVD (Wu et al., 2021). The major signaling cascades mediating the protection of acacetin against atherosclerosis are presented in Figure 3.

### 3.4 Inhibiting MF

**FIGURE 4:**
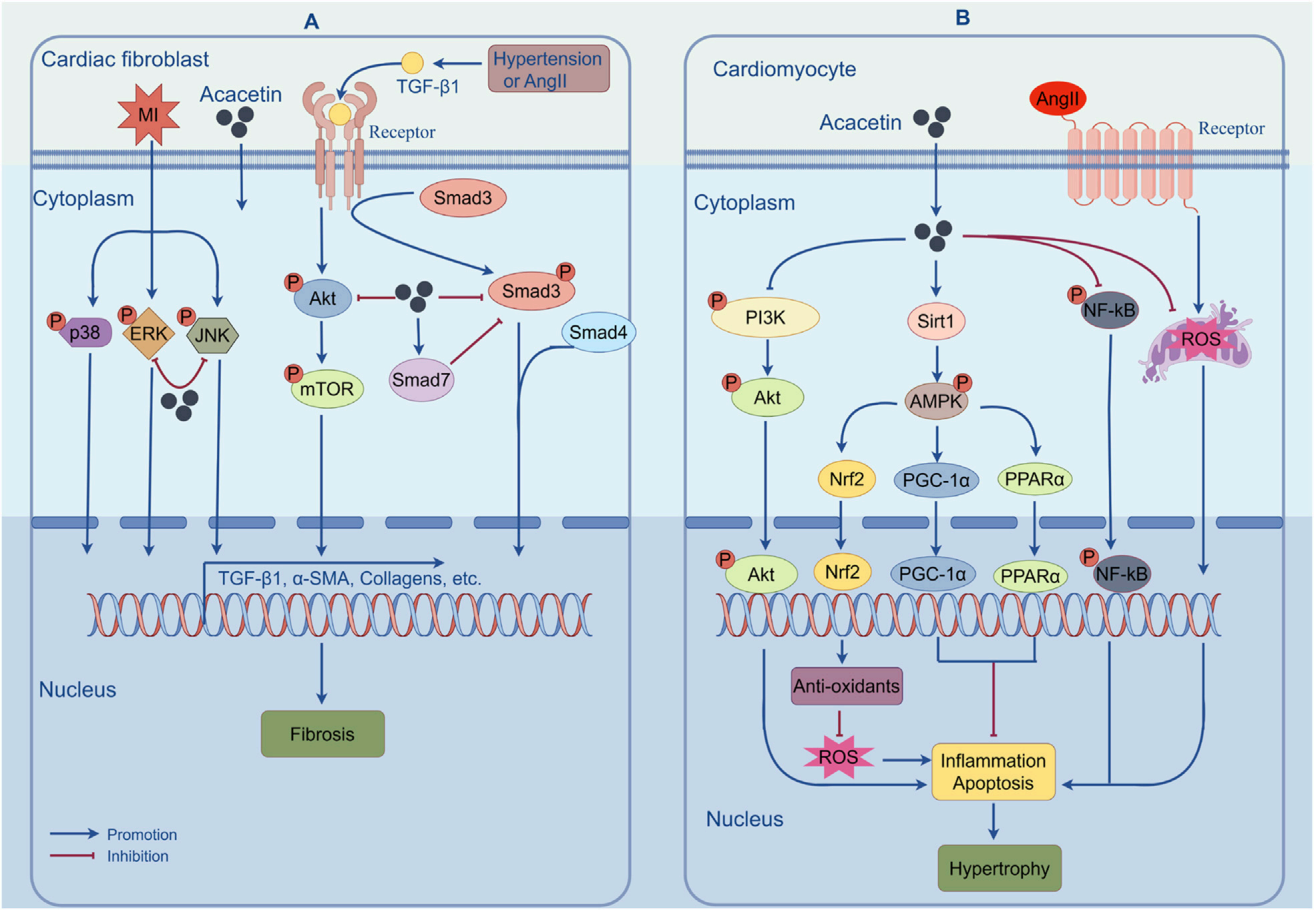
The mechanisms of action by which acacetin prevents cardiac fibrosis and hypertrophy. **(A)** Pathological stimuli such as MI, Ang II, or hypertension can induce MF by increasing the expression of fibrosis-related genes via activation of the TGF-β1/Smad3, Akt/mTOR, or MAPK signaling pathways. However, acacetin can alleviate MF by inhibiting the activation of these signaling pathways. **(B)** Acacetin can significantly counter MI- or Ang II-induced ROS production, cell apoptosis, and inflammatory responses by regulating the Sirt1/AMPK/PGC-1α, PPAR-α, Nrf2, NF-κB, or PI3K/Akt pathways, thus exerting its inhibitory effect against cardiomyocyte hypertrophy. (By Figdraw).

MF refers to the accumulation of extracellular matrix proteins (mainly collagen types I and III) in the myocardial interstitium, which can ultimately result in cardiac dysfunction and even heart failure (Kong et al., 2014). During fibrosis, activated myofibroblasts derived from resident cardiac fibroblasts are the central drivers. They not only overexpress α-smooth muscle actin but also secrete matrix proteins massively (Liu M. et al., 2021). Despite great progress in pharmacotherapy, there are still no effective therapeutic strategies for preventing MF. One previous report found that water-soluble acacetin prodrug (15 mg/kg) could remarkably improve doxorubicin (DOX)-induced MF in mice, although the underlying mechanism was not elucidated (Wu et al., 2020). In a recent report, the authors identified that acacetin (10–20 mg/kg) could significantly reduce hypertension-induced ventricular fibrosis in SHR and inhibit angiotensin II (Ang II)-stimulated proliferation, migration, and myofibroblast transformation in human cardiac fibroblasts, implying that acacetin could serve as a promising therapeutic agent for MF (Li et al., 2023). Further mechanistic analysis indicated that, as shown in Figure 4A, the inhibitory effect of acacetin on hypertension-induced MF may involve regulating the TGF-β1/Smad3 and Akt/mTOR cascades. In addition, Chang et al. showed that acacetin (10 mg/kg) could alleviate MI-induced cardiac fibrosis in mice probably by blocking the MAPK signaling pathway (Figure 4A) (Chang et al., 2017).

### 3.5 Preventing cardiac hypertrophy

Cardiac hypertrophy is initially a compensatory response of the myocardium to diverse pathological stresses such as pressure or volume overload, but prolonged or sustained stimuli induce pathological hypertrophy and can result in cardiac remodeling, heart failure, and even sudden cardiac death (Ba et al., 2019; Oka et al., 2014). Cardiac hypertrophy is commonly defined as cardiomyocyte enlargement, enhanced protein synthesis, cytoskeletal remodeling, and fibrosis development (Zhang et al., 2021). Although multiple molecular mechanisms have been identified to mediate the process of cardiac hypertrophy, effective treatment for improving cardiac hypertrophy is still lacking.

In an *in vivo* study, acacetin (10 mg/kg) was shown to alleviate post-MI cardiac hypertrophy in mice by inhibiting the PI3K/Akt signaling pathway, implying that acacetin may produce a protective effect against cardiac hypertrophy (Chang et al., 2017). The important role of cardiomyocyte apoptosis in the transition from cardiac hypertrophy to heart failure cannot be overemphasized. In this study, the authors also suggested that acacetin significantly reduced post-MI cardiomyocyte apoptosis by inhibiting p-p65, Bax, and cleaved caspase-3 expression, thereby preventing cardiac hypertrophy progression to heart failure (Chang et al., 2017). Both *in vivo* and *in vitro* studies have further elucidated the effects and mechanisms of acacetin on cardiac hypertrophy (Cui et al., 2022). The results indicated that acacetin (0.3–3 μM *in vitro;* 10 mg/kg *in vivo*) effectively attenuated Ang II-induced cardiomyocyte hypertrophy *in vitro* and abdominal aortic constriction-induced cardiac hypertrophy *in vivo* through activating the Sirt1/AMPK/PGC-1α pathway, indicating its potential for the prevention and treatment of cardiac hypertrophy. The effects and mechanisms of acacetin in protecting against cardiac hypertrophy are shown in Figure 4B.

### 3.6 Anti-cardiac senescence

**FIGURE 5:**
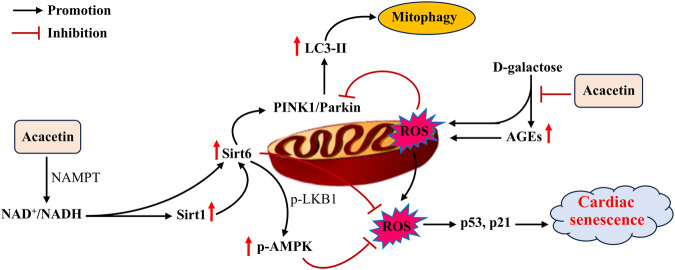
The potential mechanisms of acacetin in inhibiting D-galactose-induced cardiac senescence. D-galactose promotes advanced glycation end products (AGEs) and ROS production while suppressing mitophagy, thus leading to the formation of cardiac senescence. In addition to reducing AGEs, acacetin activates the Sirt1-mediated Sirt6/AMPK signaling cascade to enhance mitochondrial autophagy and prevent ROS production, thereby preserving mitochondrial function and improving D-galactose-induced cardiac senescence [adapted from Hong et al. (2021)].

Aging is deemed to be a primary independent risk factor for CVD (Ren and Zhang, 2018). During aging, deterioration in heart structure and function makes the heart more vulnerable to stress (Boyle et al., 2011; Shimizu and Minamino, 2019). Several molecular mechanisms, including mitochondrial dysfunction, reduced autophagy, telomere attrition, increased oxidative stress, protein acetylation, and aberrant mTOR signaling, have been demonstrated to mediate cardiac aging (Shirakabe et al., 2016; Obas and Vasan, 2018). Nevertheless, there are currently no effective therapeutic methods for reversing or slowing cardiac aging. Acacetin (1–3 μM *in vitro;* 20–50 mg/kg *in vivo*) was found to activate the Sirt1/Sirt6/AMPK signaling cascade in H9C2 cells and senescent mice induced by D-galactose, thus leading to decreases in the levels of senescence-related proteins (p53 and p21) and an enhancement of mitochondrial autophagy (as indicated by increases in the levels of autophagy-related proteins PTEN-induced kinase 1 (PINK1) and Parkin E3 ubiquitin ligase). These effects ultimately attenuated cardiac senescence (Figure 5) (Hong et al., 2021). These findings reveal that acacetin is likely to become a promising agent for improving aging-related CVD. Nevertheless, the effects of acacetin in protecting against cardiac senescence and its potential mechanisms require further investigation.

### 3.7 Improvement of DCM

Diabetes mellitus, a serious metabolic disorder with an increasing prevalence, can lead to a wide range of complications. Diabetes induce abnormalities of cardiac structure and function independent of other conventional pathological conditions, like hypertension, valvular heart disease, and coronary artery disease, known as DCM (Jin et al., 2022). DCM is defined by adverse cardiac remodeling, dysregulation in diastolic and systolic function, and eventual progression to heart failure (Gollmer et al., 2020). Despite extensive research on DCM, there is still no specific therapy for DCM.

**FIGURE 6:**
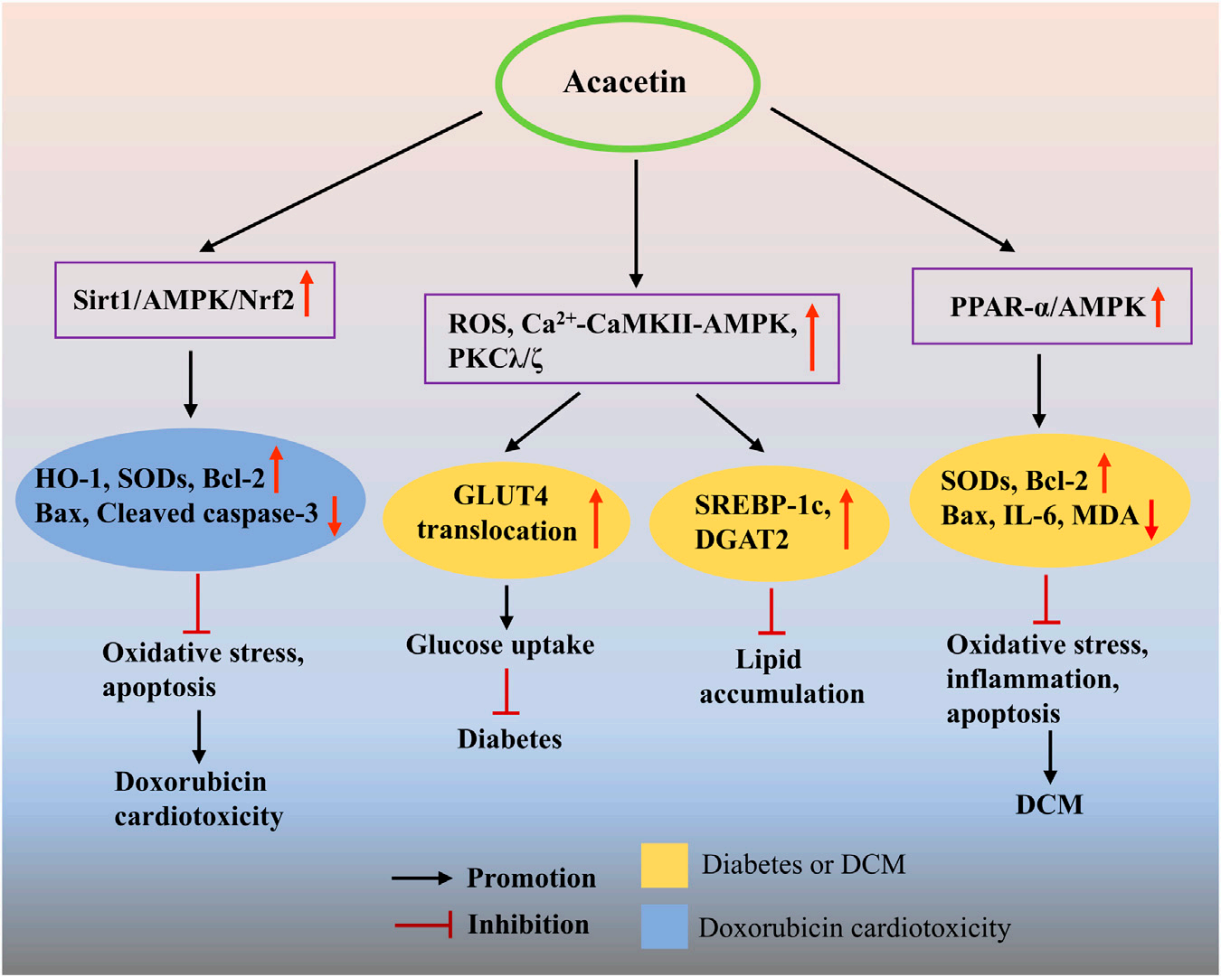
The potential mechanisms of acacetin in preventing DCM and drug-induced cardiotoxicity. Acacetin can promote glucose uptake and reduce oleic acid-induced lipid accumulation by activating the Ca^2+^-CaMKII-AMPK and PKCλ/ζ pathways, while increasing ROS production, thus exhibiting anti-diabetic properties. Besides, acacetin exerts antioxidant, anti-apoptotic, and anti-apoptotic effects by activating the PPAR-α/AMPK signaling pathway, thereby showing its therapeutic potential against DCM. In addition, acacetin can significantly alleviate DOX-induced cardiotoxicity *in vivo* and *in vitro* by inhibiting oxidative stress and cardiomyocyte apoptosis via the activation of Sirt1/AMPK/Nrf2 signaling.

In a preclinical study, it was found that acacetin (3 and 31.6 mg/kg), as a major compound extracted from the edible plant Anoda cristata, exerted a significant hypoglycemic effect in nicotinamide-streptozotocin (STZ)-induced hyperglycemic mice (Juárez-Reyes et al., 2015), which might be in association with acacetin’s antioxidant properties and PPAR-activating activities (Matin et al., 2009). Using computational chemistry approaches, researchers found that acacetin could inhibit the catalytic activity of aldose reductase by stably binding to its catalytic site and forming hydrogen bonds with the tyrosine residue at position 48, thus likely developing into a novel anti-diabetic agent (Manivannan et al., 2015). In an *in vitro* study, acacetin (20–40 μM) promoted glucose uptake in cultured L6 muscle cells by enhancing glucose transporter type 4 translocation through activation of the cytosolic free Ca^2+^-calcium/calmodulin-dependent protein kinase II (CaMKII)-AMPK and atypical protein kinase C (PKC) λ/ζ pathways, as well as promotion of intracellular ROS production, indicating that an insulin-independent mechanism may be involved in acacetin’s anti-diabetic property (Figure 6) (Kwon et al., 2020). Additionally, the study also found that acacetin (10–40 μM) inhibited oleic acid-evoked lipid deposition and increased glucose uptake in HepG2 cells, both of which were mediated by activating the AMPK signaling pathway. The above studies indicate that acacetin may be used as a novel anti-diabetic agent. To further explore the role played by acacetin in DCM and the underlying mechanism, Song et al. used high glucose-treated cardiomyocytes and STZ-induced DCM rats as an *in vitro* and *in vivo* model, respectively, and demonstrated a new pharmacological application of acacetin for the treatment of DCM (Song et al., 2022). They found that, *in vitro*, acacetin (3 μM) markedly prevented the elevation of Bax protein and the decrease of antioxidant proteins SODs induced by high glucose. *In vivo*, acacetin prodrug (10 mg/kg) obviously ameliorated cardiac dysfunction and ventricular fibrosis and inhibited the elevation of serum MDA, IL-6, and Ang II levels, as well as cardiac IL-6 and Bax expression, while preventing the reduction of serum SOD activity. These results implied that the protective effects of acacetin on DCM may be associated with its antioxidant, anti-inflammatory, and anti-apoptotic activities. Besides, they also revealed that the major mechanism by which acacetin improves DCM might be due to activating the PPAR-α/AMPK signaling pathway (Figure 6).

### 3.8 Attenuation of drug-induced cardiotoxicity

Since its clinical introduction, DOX has become one of the most effective chemotherapeutic agents for the management of various cancers, including lung cancer, breast cancer, Hodgkin’s lymphoma, acute leukemia, and others (Carvalho et al., 2009). Nevertheless, the clinical application of DOX has been limited due to its serious cardiotoxic side effects, which can contribute to chronic, progressive, and potentially life-threatening cardiomyopathy (Nebigil and Désaubry, 2018; Mitry and Edwards, 2016). Studies have shown that multiple mechanisms, including oxidative stress, mitochondrial dysfunction, calcium overload, and apoptosis in cardiomyocytes, play an instrumental role in DOX-stimulated cardiotoxicity (Ghigo et al., 2016; Wu B. B. et al., 2022). Despite this, there is still a lack of effective treatments to attenuate DOX-induced cardiomyopathy.

At present, there are very few reports available on the effects of acacetin on drug-induced cardiotoxicity. However, in a recent study, acacetin (0.3–3 μM *in vitro*; 15 mg/kg *in vivo*) notably improved DOX-evoked cardiac dysfunction and MF in mice and relieved DOX-induced cardiotoxicity in H9C2 cells through preventing oxidative stress and cardiomyocyte apoptosis via the activation of Sirt1/AMPK/Nrf2 signaling. These findings provide a basis for the future use of acacetin for the management of DOX-induced cardiomyopathy in clinical practice (Figure 6) (Wu et al., 2020).

### 3.9 Alleviating hypertension

Hypertension is a primary risk factor for CVDs. It induces vascular endothelial dysfunction, leading to diminished vascular compliance, compromised blood flow, and impaired vasodilation (Versari et al., 2009; Panza et al., 1995). The potential application of therapies focused on improving endothelial dysfunction in the prevention and treatment of hypertension should not be overlooked.

Several studies have demonstrated that acacetin has vasorelaxant effects in an *ex vivo* rat aortic ring model, although the underlying mechanisms remain unclear and require further investigation. For instance, one study reported that acacetin exhibited a tetraethylammonium chloride-insensitive vasorelaxant effect on *ex vivo* rat aortic rings (Calderone et al., 2004). In another study, acacetin, identified as one of the bioactive components of Ziziphora clinopodioides Lam., was also found to present vasodilatory activity in isolated rat aortic rings (Senejoux et al., 2012). In addition, it was indicated that acacetin, a main active compound separated from the dichloromethane-soluble extract of Agastache mexicana, had a notable vasorelaxant effect in endothelium-denuded rat aortic rings (Flores-Flores et al., 2016).

**FIGURE 7:**
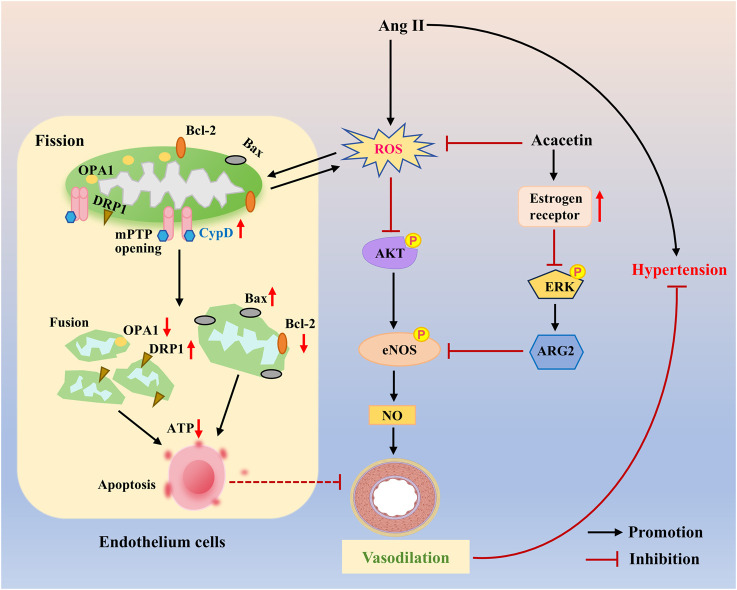
The protective mechanisms of acacetin against endothelial injury in hypertension. Acacetin enhances endothelium-dependent vasodilation by preventing ROS-evoked mitochondrial dysfunction and endothelial cell apoptosis, while activating the Akt/eNOS pathway, thereby showing anti-hypertensive effects [adapted from Li et al. (2022)]. In addition, acacetin also has estrogen-like activity, activating estrogen receptor signaling to improve endothelial dysfunction and promote vasodilation, thus reducing hypertension in SHR with insulin resistance. DRP1, dynamin-related protein 1; OPA1, optic atrophy 1; Bcl-2, B-cell lymphoma-2; CypD, cyclophilin D; mPTP, mitochondrial permeability transition pore; ARG2, arginase 2.

Estrogen acts as a vasodilator and binds to its receptors to activate downstream signaling pathways, including ERK1/2, p38 MAPK, and PI3K/Akt, thereby exerting its cardiovascular protective effects (Scott et al., 2007; Freay et al., 1997; Fardoun et al., 2020; Madak-Erdogan et al., 2008). One previous study confirmed that acacetin (25–50 mg/kg) could distinctly reduce the systolic blood pressure of insulin-resistant SHR, which might be related to the estrogen-like effect of acacetin to hamper inflammation-related cytokine expression and improve vasodilatory function in SHR with insulin resistance (Figure 7) (Wei et al., 2020). Recently, Li et al. have discovered that intraperitoneal administration of acacetin (20 mg/kg) remarkably lowered the mean arterial pressure only in SHR but not in Wistar-Kyoto (WKY) rats with normal arterial blood pressure. This effect was likely mediated by acacetin’s ability to induce endothelium-dependent vasorelaxation in rat mesenteric arteries via activation of the Akt/eNOS cascade and restoration of mitochondrial function (Figure 7) (Li et al., 2022).

## 4 Current problems and prospects

Obviously, it is certain that acacetin possesses some health benefits due to its various bioactivities. Accumulating evidence has indicated that acacetin shows therapeutic potential in multiple CVDs. However, the protective effects of acacetin against CVD mainly relies on data derived from existing animal models and *in vitro* experiments, and it is unclear whether acacetin still possesses cardiovascular protective effects in humans. Therefore, clinical studies are required to validate these preclinical findings. In addition, as a possible supplement to clinical trials, human iPSC-derived cardiovascular cells and organoids can be used as experimental models to further clarify the cardiovascular protective effects and mechanisms of acacetin.

Although the plant sources of acacetin are up to 92, most of which are perennial species and often need a long period to reproduce. Currently, few studies have been conducted on the synthesis and extraction methods of acacetin. Meanwhile, plant-derived extraction processes are costly and time-consuming. As a result, it is necessary to develop new cost-effective synthesis strategies to increase the yield of acacetin.

Some studies have reported that acacetin has the disadvantages of very poor water solubility, short half-life, rapid metabolism in various tissues, especially in the liver, and rapid decrease in blood concentration, all of which greatly limit its clinical application. Therefore, future research on acacetin should focus on how to improve its solubility and bioavailability. Of note, there are some conventional and emerging techniques and methods worth considering, such as chemical structural modification (e.g., prodrug synthesis, glycosylation), microemulsion or nanoparticle delivery formulations, carrier complexes, pharmaceutical cocrystals, and the utilization of absorption enhancers. These strategies have been developed to enhance the solubility, stability, and absorption rate of flavonoids (Zhao et al., 2019). As we know, in addition to bioavailability, toxicity evaluation is another important element of drug commercialization. However, at present, there are very limited studies on acacetin toxicity and pharmacokinetics. Therefore, in order to ensure the safety of acacetin for clinical usage, it is necessary to conduct more investigations, including Phase I clinical trials, for the establishment of safe dosage ranges that balance pharmacological efficacy with potential toxicity.

## 5 Conclusion

This article not only reviews the properties of acacetin in terms of physicochemical characteristics, biosynthesis, pharmacokinetics, biotoxicity, and biological activities but also analyzes its potential benefits in treating CVDs and related molecular mechanisms. A growing body of studies has demonstrated that acacetin can exert potent cardiovascular protection against multiple pathologies, including arrhythmia, atherosclerosis, hypertension, DCM, myocardial fibrosis and hypertrophy, cardiac I/R injury, myocardial senescence, and even drug-induced cardiotoxicity. The underlying mechanisms involve inhibiting oxidative stress, reducing inflammation, preventing cardiomyocyte apoptosis and endothelial cell injury, as well as regulating mitochondrial autophagy and lipid metabolism. Despite the low water solubility and poor bioavailability of acacetin, there are some conventional and emerging techniques and methods to solve these problems. In brief, acacetin is promising to become a novel drug candidate for preventing and treating CVDs and deserves more attention and investigations to facilitate its translation and clinical application.
